# A rare autism-associated MINT2/APBA2 mutation disrupts neurexin trafficking and synaptic function

**DOI:** 10.1038/s41598-019-42635-7

**Published:** 2019-04-15

**Authors:** Amy Y. Lin, Shawna Henry, Carsten Reissner, Christian Neupert, Connor Kenny, Markus Missler, Uwe Beffert, Angela Ho

**Affiliations:** 1 Department of Biology, Boston University, 24 Cummington Mall, Boston, MA 02215 USA; 2 Institute of Anatomy and Molecular Neurobiology, Westfälische Wilhelms-University, 48149 Münster, Germany; 3 Cluster of Excellence EXC 1003, Cells in Motion, 48149 Münster, Germany

## Abstract

MINT2/APBA2 is a synaptic adaptor protein involved in excitatory synaptic transmission. Several nonsynonymous coding variants in MINT2 have been identified in autism spectrum disorders (ASDs); however, these rare variants have not been examined functionally and the pathogenic mechanisms are unknown. Here, we examined the synaptic effects of rat Mint2 N723S mutation (equivalent to autism-linked human MINT2 N722S mutation) which targets a conserved asparagine residue in the second PDZ domain of Mint2 that binds to neurexin-1α (Nrxn1α), a presynaptic cell-adhesion protein implicated in ASDs. We show the N723S mutation impairs Nrxn1α stabilization and trafficking to the membrane while binding to Nrxn1α remains unaffected. Using time-lapse imaging in primary mouse neurons, we found that the N723S mutant had more immobile puncta at neuronal processes compared to Mint2 wild type. We therefore, reasoned that the N723S mutant may alter the co-transport of Nrxn1α at axonal processes to presynaptic terminals. Indeed, we found the N723S mutation affected Nrxn1α localization at presynaptic terminals which correlated with a decrease in Nrxn-mediated synaptogenesis and miniature event frequency in excitatory synapses. Together, our data reveal Mint2 N723S leads to neuronal dysfunction, in part due to alterations in Nrxn1α surface trafficking and synaptic function of Mint2.

## Introduction

Autism Spectrum Disorders (ASDs) comprise a heterogeneous group of neurodevelopmental disorders characterized by a complex genetic etiology and impairments in social skills, communication and repetitive behaviors. In humans, chromosome 15q11-q13 has been identified as a strong candidate region for autism susceptibility based on the frequent occurrence of chromosomal abnormalities and numerous linkage and genome-wide association studies^1,2^. The 15q11-q13 region consists of several neuronal genes including *MINT2* (also known as *APBA2* or *X11*β) which encodes for a synaptic adaptor protein and is implicated in ASDs and neuropsychiatric phenotypes based on several lines of evidence. First, *MINT2* maps to the distal portion of 15q13.1, a region commonly deleted in neurodevelopmental disorders such as Prader-Willi and Angelman syndromes and duplicated in cases of autism^3–5^. Second, several studies have independently identified copy number variants (CNVs) in *MINT2* associated with autism^6,7^. Third, seven nonsynonymous coding variants in MINT2 have been identified in rare individuals with ASDs^8^. Further, an independent study examining gene expression differences in autism brain tissue identified *MINT2* as one of four key molecules differentially coexpressed with *CNTNAP1*, *CHRM1* and *A2BP1* in brains^9^. These genes are involved in synaptic function, vesicular transport and neuronal projection suggesting a complex interplay of genes from multiple pathways associated with ASDs^9^. Lastly, Mint2 interacts directly with neurexin1α (Nrxn1α), a regulator of synaptic properties, which is strongly associated with ASDs^10–12^. Furthermore, both *MINT2* and *NRXN1* are implicated in schizophrenia^13^. The combined evidence of independent mutations and CNVs in *MINT2* and the interaction with another ASD synaptic protein makes *MINT2* a plausible candidate that plays a key role in ASD pathogenesis. However, little is known about how these rare autism-linked mutations in *MINT2* lead to ASD pathogenesis.

MINT2 belongs to a family of neuronal adaptor proteins (MINTs 1–3) that is also known as APBAs 1–3 or X11-like proteins (X11α, X11β, X11γ). They consist of a divergent N-terminus and a conserved C-terminus composed of a phosphotyrosine-binding (PTB) domain and two PDZ domains^14,15^. MINTs are evolutionary conserved and there is over 90% sequence homology between human MINT2 and rat Mint2. The functionality of the Mints is determined by their multi-domain structure and the nature of their interacting partners. In the N-terminal region, neuronal Mints 1 and 2 bind to Munc18-1, an essential synaptic fusion protein, linking Mints to synaptic vesicle exocytosis^14,16^. In the C-terminal region, the PTB domain of all three Mints binds to the amyloid precursor protein (APP) and alters APP proteolytic processing which is centrally involved in the pathogenesis of Alzheimer’s disease^17,18^. Moreover, *via* their PDZ domains, Mints bind to a number of proteins *in vitro*, including Nrxn1α^10^, presenilin^19,20^, and voltage-gated calcium channels^21^. Double knockouts of Mints 1 and 2 in mice showed a 20% survival rate and surviving mice at 3–4 weeks of age exhibit decreased synaptic strength and a decline in presynaptic neurotransmitter release machinery^16^. Furthermore, knockout of a single Mint2 allele in mouse displays a deficit in social interaction supporting the role of Mint2 in emotional and social development^22^. Altogether, these results suggest that Mint proteins are important for synaptic function and that loss of function leads to phenotypes consistent with those observed in ASDs.

Here, we surveyed the seven nonsynonymous *MINT2* coding variants identified by Babatz *et al*., 2009 to assess whether these rare individual variants in *MINT2* affect protein expression. Of the seven MINT2 variants, we focused on the rat Mint2 N723S mutation, equivalent to human MINT2 N722S, and found it profoundly affected Nrxn1α stabilization and surface trafficking. We found that the N723S mutation decreased Nrxn1α localization in axons and the presynaptic terminals of primary mouse neurons leading to alterations in Nrxn-mediated synaptogenesis and synaptic function.

## Results

### Mint2 N723S mutation affects Nrxn1 stabilization and membrane trafficking

**Figure 1:**
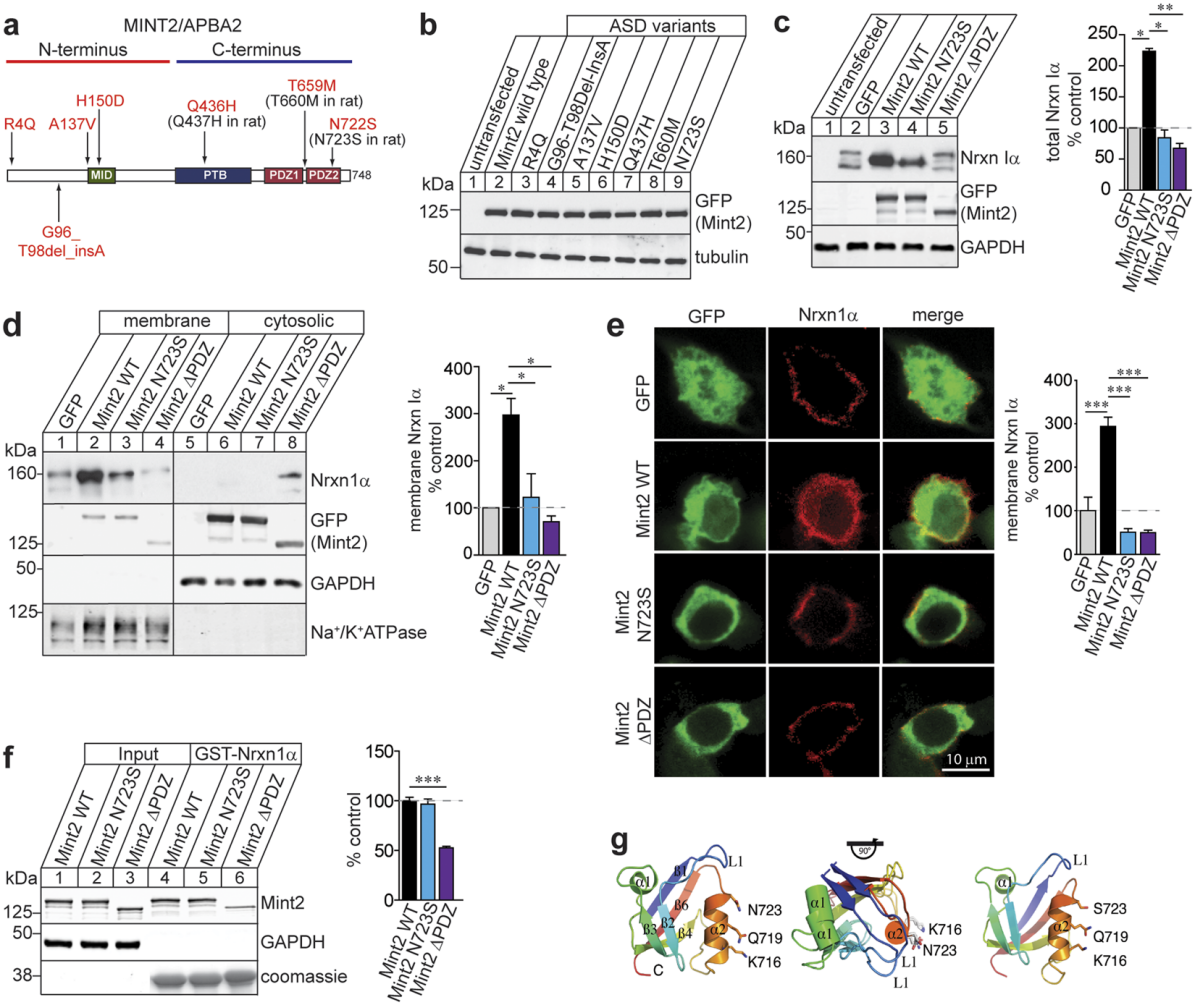
Mint2 N723S mutation decreases Nrxn1α membrane trafficking in HEK293T cells. (**a**) Structure of human MINT2 and locations of MINT2 ASD variants. (**b**) Representative immunoblots of protein levels from total cell lysates of HEK293T cells transfected with Mint2 WT or Mint2 ASD variants. Tubulin served as loading control on the same blot. The representative images were cropped and shown in this figure. Whole gel images and quantification are presented in Supplementary Fig. S1a,b, respectively. **(c)** Representative immunoblots of protein levels from total cell lysates of HEK293T cells co-transfected with Nrxn1α and either GFP (control), GFP-tagged Mint2 WT, N723S or ΔPDZ. GAPDH served as loading control on the same blot. The representative images were cropped and shown in this figure. Whole gel images are presented in Supplementary Fig. S1c. Quantification of total Nrxn1α expressed as percent control and is based on three independent experiments. **(d)** Western blot analysis and quantification of membrane Nrxn1α levels expressed as percent control from HEK293T cells co-transfected with Nrxn1α and either GFP (control), GFP-tagged Mint2 WT, N723S or ΔPDZ and subjected to subcellular fractionation. GAPDH and Na^+^/K^+^ ATPase were used as cytosolic and membrane markers, respectively. The representative images from n = 3 independent experiments were cropped and shown in this figure. Whole gel images are presented in Supplementary Fig. S1d. **(e)** Representative images of HEK293T cells co-transfected with Flag-Nrxn1α together with GFP (control), GFP-tagged Mint2 WT, N723S or ΔPDZ. Quantification of cell surface Nrxn1α expressed as percent control. GFP, n = 17; WT, n = 31; N723S, n = 33; ΔPDZ, n = 22 total number of cells analyzed from at least 2 coverslips per condition with n = 2 independent experiments/cultures. **(f)** Western blot analysis and quantification of Mint2 bound to GST-Nrxn1α fusion protein expressed as percent control (Mint2 WT) from HEK293T cells transfected with Mint2 WT, N723S or ΔPDZ. Coomassie stained gel of GST-tagged Nrxn1α protein. The representative images from n = 3 independent experiments were cropped and shown in this figure. Whole gel images are presented in Supplementary Fig. S1e. **(g)** The second PDZ domain of Mint2 WT (PDB_ID: 3SUZ) consists of six β-strands and two α-helices (left). Mint2 PDZ2 is highly dynamic showing a relative rigid-body motion around a fixed α2 (middle). Mint2 N723S mutation does not change the conformation of α2 and the Nrxn binding pocket between α2 and β2 (right). Data represents as means ± SEM and statistical significance was evaluated using one-way ANOVA. All determinations, *p < 0.05; ***p < 0.0005 (see text). Scale bar = 10 μm.

Seven rare nonsynonymous coding variants spanning human *MINT2* have been genetically-linked to ASD patients (Fig. 1a)^8^. Five of the seven *MINT2* mutations were predicted to affect protein function (R4Q, A137V, H150D, Q436H, and T659M)^8^; however, these mutants have not been examined functionally. To determine whether any of these MINT2 variants affect protein expression, we generated GFP-Mint2 mutants bearing the seven human ASD MINT2 mutations individually within rat Mint2 cDNA, since the amino acid residues are highly conserved between human and rat Mint2. We examined whether Mint2 mutants were properly expressed *in vitro* by transfecting Mint2 wild type (WT) or mutants in HEK293T cells and immunoblotting for the GFP-tagged Mint2 protein (Fig. 1b and Supplementary Fig. S1a). The protein expression profile of Mint2 showed that Mint2 mutants expressed at similar levels compared to Mint2 WT, suggesting that these Mint2 mutants did not disrupt Mint2 protein expression (Supplementary Fig. S1b).

Because *NRXN1* is a strong candidate gene associated with ASDs^11,12^ and interacts directly with Mint2^10^, we next tested whether any of the Mint2 variants affected Nrxn1 protein stability. We co-transfected Nrxn1α with either GFP (control), GFP-tagged Mint2 WT or mutants in HEK293T cells and consistently found the N723S mutation (equivalent to the autism-linked human MINT2 N722S mutation) alter Nrxn1α levels compared to Mint2 WT. Mint2 WT increased Nrxn1α protein levels compared to GFP control [one-way ANOVA, F_(3,14)_ = 7.499, p = 0.0233] (Fig. 1c and Supplementary Fig. S1c). We found N723S decreased Nrxn1α protein levels compared to Mint2 WT, possibly affecting Nrxn1α protein stability [one-way ANOVA, F_(3,14)_ = 7.499, p = 0.0067] (Fig. 1c). In addition, co-expression of Nrxn1α with Mint2 ΔPDZ (C-terminal truncation lacking both Mint2 PDZ domains), a mutant that affects Nrxn1α binding, decreased Nrxn1α levels compared to Mint2 WT [one-way ANOVA, F_(3,14)_ = 7.499, p = 0.0043], suggesting the Mint2-Nrxn1α interaction is critical for Nrxn1α stability. Because Mint2 N723S disrupts a conserved asparagine residue in the second PDZ domain of Mint2 (a domain which binds to Nrxns) showed a robust effect on Nrxn1α protein stability, we focused on the pathophysiological effect of this single mutation and how Mint2 N723S may be associated with ASDs.

The apparent increase in abundance of Nrxn1α protein induced by Mint2 WT expression is consistent with the association of Nrxn1α with Mint2 in the secretory pathway and may indicate the Mint2-Nrxn1α interaction is important for targeting Nrxn1α to the cell surface. To determine whether this association increased the surface levels of Nrxn1α, we subjected HEK293T cells to subcellular fractionation following co-transfection of Nrxn1α with either GFP (control), GFP-tagged Mint2 WT, N723S or ΔPDZ. Mint2 WT greatly increased Nrxn1α by 3-fold at the membrane (defined as material that is soluble in 2% Triton X-100/2% NP40) compared to GFP [one-way ANOVA, F_(3,4)_ = 10.80, p = 0.0246], whereas Nrxn1α levels associated with N723S and ΔPDZ was significantly decreased compared to Mint2 WT [one-way ANOVA, F_(3,4)_ = 10.80, p = 0.0365, p = 0.0153, respectively] (Fig. 1d and Supplementary Fig. S1d). Quantification of membrane Nrxn1α is defined as Nrxn1α protein levels in membrane fraction normalized to membrane marker Na^+^/K^+^ ATPase levels and expressed as percent GFP control. We detected cytosolic Nrxn1α levels exclusively in the Mint2 ΔPDZ mutant suggesting Nrxn1α is possibly sequestered to a non-functional, cytosolic compartment (Fig. 1d).

To corroborate the biochemical analysis, we performed a live cell surface staining to quantify the relative amount of surface Nrxn1α in HEK293T cells that were co-transfected with Flag-Nrxn1α and either GFP (control), GFP-tagged Mint2 WT, N723S or ΔPDZ. Cells were incubated with ice-cold extracellular N-terminal Flag antibody to label surface-bound Flag-Nrxn1α for 30 min at 4 °C to prevent internalization. Following ice-cold PBS washes, cells were fixed and incubated with a fluorescent-conjugated secondary antibody. The level of surface Nrxn1α was markedly different between Mint2 WT and GFP control. Mint2 WT showed a 3-fold increase in cell surface Nrxn1α compared with GFP control [one-way ANOVA, F_(3,92)_ = 51.08, p < 0.0001] (Fig. 1e). Consistent with the biochemical analysis, both N723S and ΔPDZ mutations reduced the level of surface Nrxn1α compared to Mint2 WT in HEK293T cells [one-way ANOVA, F_(3,92)_ = 51.08, p < 0.0001 for both]. Together, these data indicate the Mint2-Nrxn1α interaction is important for targeting Nrxn1α to the cell surface and the steady-state surface expression of Nrxn1α is severely affected by the N723S mutation.

To examine whether the N723S mutation affects Nrxn1α binding, the cytoplasmic tail of Nrxn1α was immobilized as a GST-fusion protein to examine whether Nrxn1α pulls down full-length Mint2 WT or N723S mutation that were overexpressed from HEK293T cell lysates (Fig. 1f and Supplementary Fig. S1e). We found Mint2 WT bound to Nrxn1α whereas the Mint2-ΔPDZ dramatically reduced its binding to Nrxn1α by 47.5% compared to Mint2 WT [one-way ANOVA, F_(2,5)_ = 131.9, p < 0.0001]. The fact that Mint2-ΔPDZ only reduced Nrxn1α binding by 47.5% suggests that the N-terminus of Mint2 or other factors of Mint2 such as other binding partners of Mint2 could participate in the interaction between Mint2 and Nrnx1α. Meanwhile, the N723S mutation did not disrupt binding to Nrxn1α. To examine this further, we predicted the effect of Mint2 N723S mutation on the Mint2 structure by molecular dynamic simulation. Previous studies have shown the second PDZ domain of Mint2 consists of six β-strands and two α-helices^23^, and this structure is highly dynamic showing a relatively rigid body motion around a fixed α2 helix (Fig. 1g). Molecular dynamic calculations predict the N723S mutation does not change the α2 helix conformation of the second PDZ domain of Mint2, suggesting the mutation likely does not modify the binding pocket to which Nrxn1α binds. Together, results from both the pull-down experiment and the molecular dynamic simulation suggest that Mint2 N723S is unlikely disrupting binding to Nrxn1α. Instead, we found that the Mint2 N723S mutation affects Nrxn1α stabilization and membrane trafficking.

### Mint2 N723S leads to an increased number of immobile puncta at the Golgi apparatus and neuronal processes

**Figure 2:**
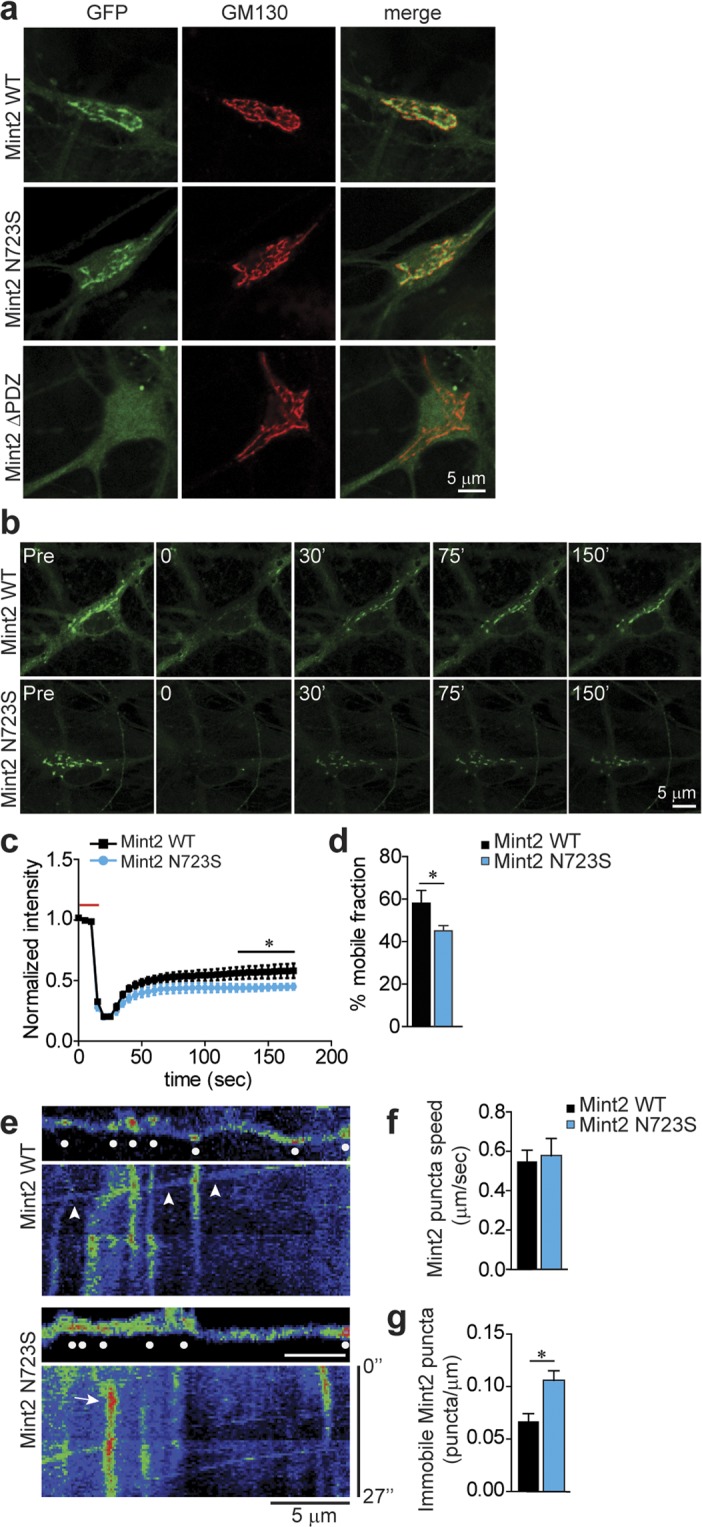
Mint2 N723S leads to an increased number of immobile puncta in the Golgi apparatus and neuronal processes in primary mouse neurons. **(a)** Mint2 localizes to the Golgi in primary mouse hippocampal neurons. Representative images of hippocampal neurons infected with lentiviral GFP-Mint2 WT, GFP-Mint2 N723S or GFP-Mint2 ΔPDZ and immunostained with GM130 (a Golgi maker) at 14 DIV. Both Mint2 WT and N723S co-localized with GM130 but not Mint2 ΔPDZ (represented as yellow in merged image). N = 12–15 cells from at least three coverslips that was analyzed for each group from three independent experiments/cultures. **(b)** Time-lapse FRAP imaging of GFP-tagged Mint2 WT and N723S at the Golgi. Regions encompassing the Golgi were photobleached using 3 passes (15 s total) of 100% laser power, which reduced the initial fluorescence intensity to 25%. Recovery fluorescence was acquired with 1% laser power and imaged every 5 s for 150 s. **(c)** Normalized intensity of GFP-tagged Mint2 WT and N723S signal during FRAP. Horizontal red bar indicates the period of photobleaching on graph. WT, n = 8 and N723S, n = 10 total number of cells assessed. **(d)** Percentage of Mint2 that is mobile in the Golgi, as measured by percentage of fluorescence that recovers after photobleaching at 170 s. **(e)** Trafficking and localization of Mint2 to neuronal processes. Top, Representative images of GFP-tagged Mint2 WT and N723S puncta in segments of processes. Bottom, corresponding kymographs for the segments. Diagonal bands indicate mobile Mint2 puncta (arrowheads) and vertical bands indicate immobile Mint2 puncta (arrow). Dotted white circles indicate puncta. **(f)** Bar graph of puncta speed showed no change. **(g)** Mint2 N723S has more immobile puncta compared to the WT. WT, n = 19 and N723S, n = 20 total number of processes assessed. Data represents as means ± SEM and statistical significance was evaluated using unpaired Student’s *t-*test. All determinations, *p < 0.05 (see text). Scale bar = 5 μm.

Previous studies have shown Mint1 is a mobile protein in primary rat cortical neurons and functions at the Golgi to control polarized trafficking of axonal membrane proteins including APP^24,25^. Because Mints are localized to the Golgi in primary murine neurons^20^, we tested whether GFP-tagged Mint2 N723S or Mint2 ΔPDZ altered the localization of Mint2 at the Golgi in primary mouse neurons by immunolabeling with the Golgi marker GM130. Both GFP-Mint2 WT and N723S were predominantly localized in a perinuclear compartment that overlapped with GM130; however, GFP-Mint2 ΔPDZ localization was largely diffuse and did not co-localize with GM130 at the Golgi (Fig. 2a).

Because Mints are localized to the Golgi and we did not observe any localization changes with the N723S mutation, we wanted to test whether the N723S mutant affects protein dynamics at the Golgi to control and traffic membrane proteins. We therefore performed fluorescence recovery after photobleaching (FRAP) to determine the kinetics of Mint2 protein in live primary mouse neurons infected with GFP-tagged Mint2 WT or N723S lentivirus at 3 DIV and analyzed at 14 DIV (Fig. 2b). Upon expression of the GFP-tagged Mint2 constructs, neuronal localization of Mint2 was apparent in punctate-like clusters at the Golgi. We found both WT and N723S fluorescence signal effectively depleted by photobleaching and partially recovered at the Golgi as would be expected for a mobile protein (Fig. 2c). However, the fluorescence recovery pattern was not the same; 58% of WT recovered within 170 s as compared with 45% of N723S [unpaired Student’s *t-*test, p < 0.044] suggesting N723S had a larger immobile fraction compared to Mint2 WT (Fig. 2d). However, the time at which 50% of equilibrium fluorescence level is recovered (τ_1/2_) from WT (23.2 ± 7.2 sec) and N723S (27.3 ± 12.4 sec) at the Golgi was not different [unpaired Student’s *t-*test, p < 0.775] suggesting Mint2 N723S did not alter Mint2 transport kinetics.

Since the N723S mutation resulted in more immobile puncta at the Golgi, we reasoned that it may also alter Mint2 puncta kinetics in neuronal processes. We therefore examined mobility changes of GFP-tagged Mint2 WT and N723S puncta in primary mouse neuronal processes at 14 DIV. We used kymograph analysis to plot the fluorescence intensity of WT and N723S mutant across distance and time. Mobile and stationary puncta could be visualized with stationary puncta seen as vertical bands and mobile puncta indicated by diagonal bands within the kymograph (Fig. 2e). Consistent with the FRAP data, while the speed of the mobile Mint2 puncta was similar in both WT (0.55 ± 0.06 μm/sec) and N723S mutant (0.57 ± 0.09 μm/sec) [unpaired Student’s *t-*test, p < 0.770] (Fig. 2f), the percentage of immobile Mint2 puncta was greater in the N723S mutant [unpaired Student’s *t-*test, p < 0.0023] (Fig. 2g). Overall, our data suggest that Mint2 N723S leads to an increased number of immobile puncta in neuronal processes.

### Mint2 N723S alters Nrxn1α axonal localization to presynaptic terminals

**Figure 3:**
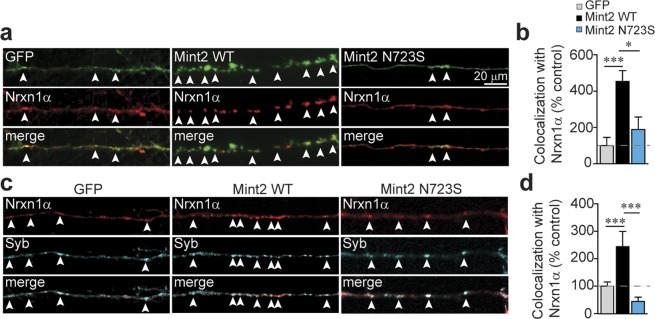
Mint2 N723S affects axonal localization of Nrxn1α to synapses. **(a)** Representative images of axonal segments of hippocampal neurons co-transfected with mCherry-Nrxn1α and GFP-tagged Mint2 WT or N723S at 4–6 DIV and analyzed at 7–10 DIV. **(b)** Quantification of co-localization (represented by arrowheads and yellow in the merged images in *a*) expressed as percent control between Nrxn1α and GFP (control), WT or N723S. GFP; n = 14, WT, n = 12; or Mint2 N723S, n = 10 total of axons from at least two coverslips that was analyzed from three independent experiments/cultures. **(c)** Representative images of hippocampal neurons co-transfected with mCherry-Nrxn1α and GFP-tagged Mint2 WT or N723S at 4–6 DIV and immunostained for presynaptic anti-synaptobrevin - syb (cyan) marker at 12–14 DIV. **(d)** Quantification of co-localization (represented by arrowheads and white in merged images in *c*) expressed as percent control between Nrxn1α and Syb. GFP; n = 23, WT, n = 16; or N723S, n = 21 total of processes analyzed. For 3b and 3d, “n” represents single axons from at least two coverslips that was analyzed from three independent experiments/cultures. Data represents as means ± SEM and statistical significance was evaluated using unpaired Student’s *t*-test. All determinations, **p < 0.005; ***p < 0.0005 (see text). Scale bar = 20 μm.

Nrxns are delivered to the presynaptic membrane by a polarized and regulated targeting process that involves their C-terminal sequences binding to different trafficking partners through the secretory pathway^26,27^. We hypothesize that Mint2 functions at the Golgi to control polarized axonal trafficking of Nrxn1α to the synapse. Our results indicate that both Mint2 WT and N723S interact with the C-terminal sequences of Nrxn1α similarly; however, we showed that N723S mutation profoundly affected Nrxn1α stabilization and trafficking to the membrane (Fig. 1). Since we found the N723S leads to an increased number of immobile puncta in neuronal processes (Fig. 2), we reasoned that the N723S mutant may alter the co-transport of Nrxn1α at axonal processes to presynaptic terminals. To determine whether Nrxn1α was co-transported with Mint2 at axonal processes and to examine whether N723S interferes with Nrxn1α co-transport in primary mouse hippocampal neurons, mCherry-Nrxn1α was co-transfected with either GFP (control), GFP-tagged Mint2 WT or N723S at 4–6 DIV and analyzed at 7–10 DIV, because we were interested in the early events of Nrxn1α trafficking on axons before synaptogenesis. We quantified the percentage of the total Nrxn1α puncta that co-labeled with GFP in axonal processes which is indicated as merge (yellow to represent co-localization) (Fig. 3a). We observed a high degree of co-localization between transfected mCherry-Nrxn1α and GFP-tagged Mint2 WT compared to GFP control [one-way ANOVA, F_(2,33)_ = 11.15, p < 0.0001] (Fig. 3b). However, the co-localization signal was not as robust when neurons were co-transfected with mCherry-Nrxn1α and the N723S mutation compared to Mint2 WT [one-way ANOVA, F_(2,33)_ = 11.15, p = 0.0063] suggesting the possibility that less Nrxn1α is being trafficked to the presynaptic membrane *via* Mint2.

To determine whether the tagged mCherry-Nrxn1α was successfully transported by Mint2 to presynaptic sites in the axonal plasma membrane, primary mouse hippocampal neurons were co-transfected with mCherry-Nrxn1α and either GFP control, GFP-tagged Mint2 WT or N723S at 4–6 DIV and analyzed at 12–14 DIV when synaptic contacts are maturing. We performed immunofluorescence analysis using the presynaptic marker, synaptobrevin (Syb) to examine Nrxn1α trafficking to the synapse (Fig. 3c). Nrxn1α showed a high degree of co-localization with synaptobrevin in neurons transfected with Mint2 WT compared to both GFP control and N723S [one-way ANOVA, F_(2,57)_ = 110158, p < 0.0001 for both] (Fig. 3d). Altogether, these results suggest that Mint2 WT induced Nrxn1α axonal localization to presynaptic terminals whereas the N723S mutation was unable to do so. While these experiments measure alterations to overexpression of mCherry-tagged Nrxn1α, it remains uncertain whether Mint2 have direct effects on endogenous Nrxn1α.

### Mint2 N723S disrupts Nrxn-mediated synaptogenesis in a heterologous synapse formation assay

**Figure 4:**
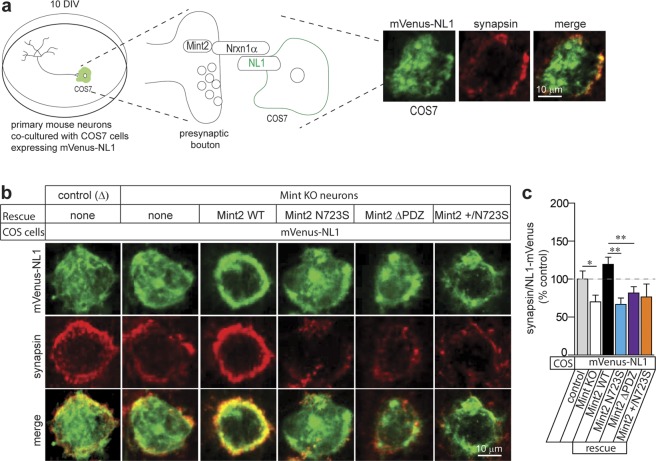
Mint2 N723S decreases Nrxn-mediated synapse formation. **(a)** Illustration shows COS7 cells (green) expressing mVenus-NL1 were seeded over dissociated primary mouse hippocampal neurons (black) at 10 DIV and co-cultured for 2 days before analysis. Synaptic contact between neuronal axon and COS7 cells are enlarged which shows a synaptic complex of Mint2-Nrxn1α (presynaptic) with NL1. Sites of cell contact with neuronal axons are analyzed for the recruitment of presynaptic marker, synapsin. The images on the right depict representative immunofluorescence image of COS7 cell expressing mVenus-NL1 in the mixed culture where green represents mVenus, red is synapsin and yellow is merge indicating co-localization. Scale bar = 10 μm. **(b)** COS cells expressing mVenus-NL1 were co-cultured with control or Mint KO neurons where the latter were additionally rescued with lentiviruses expressing Mint2 WT, N723S, ΔPDZ or heterozygous +/N723S. Scale bar = 10 μm. **(c)** Quantification of synapse formation expressed as percent control was determined by the co-localization (yellow in the merged images) of the presynaptic synapsin (red) with postsynaptic mVenus- Nlgn1 (green) expression. Control, n = 31; KO, n = 30; WT, n = 26; N723S, n = 34; ΔPDZ, n = 35; +/N723S, n = 29 total number of cells analyzed. “n” represents multiple cells from at least 2 coverslips per condition and analyzed from three independent experiments/cultures. Data represents as means ± SEM and statistical significance was evaluated using one-way ANOVA. All determinations, *p < 0.05; ***p < 0.0005 (see text).

Based on our data, we reasoned that Mint2 may function as a mobile protein at the Golgi to control polarized trafficking of Nrxn1α to the presynaptic membrane. Trafficking of Nrxn1α to the presynaptic site is essential for normal synapse formation and function and the N723S mutation may disrupt Nrxn-mediated synaptogenesis (Figs 2 and 3). Previous studies have shown that Nrxns can bind to postsynaptic cell-adhesion Neuroligin (NL1) and participate in organizing synaptic structures^28,29^. For example, expression of NL1 in a non-neuronal cell induces synapse formation by co-cultured neurons, referred to as heterologous synapse formation that involves the engagement of presynaptic Nrxns^30,31^. To test whether Mint2 contributes in synaptic formation dependent on Nrxn-NL1 binding, we used an established heterologous neuron-COS7 co-culture assay where NL1 is expressed in non-neuronal COS7 cells to induce the formation of presynaptic specifications with co-cultured neurons^30,32^ (Fig. 4a). Because Mints 1–3 have redundant functions in the mice and to examine the direct effects of Mint2 N723S mutant in neurons^16^, we used a conditional knockout (KO) mouse line that is homozygous for Mint1 and 3 KO and floxed for Mint2 (*Mint1*^−/−^*; fMint2//fMint2; Mint3*^−/−^). Primary hippocampal neurons from *Mint1*^−/−^*; fMint2//fMint2; Mint3*^−/−^ were infected at the day of plating with lentiviruses carrying *cre* recombinase to knockout Mint2, and rescued with lentivirus carrying GFP-tagged Mint2 WT, N723S, ΔPDZ or equimolar amounts of Mint2 WT and N723S referred to as +/N723S since these Mint2 ASD variants were inherited from unaffected parents. In addition, a mutant *cre* recombinase served as control (Δ). We plated COS7 cells transfected with mVenus-NL1 onto primary hippocampal neurons at 10 DIV to induce synapse formation indicated by presynaptic synapsin staining clustered on the NL1 expressing COS-7 cells (Fig. 4a,b). Quantification of synapse formation was expressed as percent control (Δ) that was determined by the co-localization (yellow in the merged images) of the presynaptic synapsin (red) and postsynaptic mVenus-NL1 (green) expression. We found Mint KO neurons impaired heterologous synapse formation compared to control infected neurons that contain Mint2 [one way ANOVA, F_(4, 15)_ = 6.042, p = 0.0153] (Fig. 4c). Infection of lentiviral Mint2 WT was able to rescue Nrxn-mediated heterologous synapse formation in Mint KO neurons. However, we found N723S was not able to rescue the presynaptic clustering of synapsin in Mint KO neurons compared to Mint2 WT [one way ANOVA, F_(4, 15)_ = 6.042, p = 0.0042], suggesting that the N723S mutant may not efficiently facilitate the transport of Nrxn1α to the presynaptic membrane to rescue heterologous synapse formation (Fig. 4c). Similarly, the Mint2-ΔPDZ mutant (which decreased binding to the C-terminus of Nrxn1α, Fig. 1f), also impaired rescue of heterologous synapse formation in Mint KO neurons compared to Mint2 WT [one way ANOVA, F_(4, 15)_ = 6.042, p = 0.0027]. When we examined the heterozygous + /N723S mutant, the resulting mutant was not able to fully rescue heterologous synapse formation, although this was not significant compared to Mint2 WT [one way ANOVA, F_(4, 15)_ = 6.042, p = 0.3422]. These results demonstrate that the Mint2-Nrxn1α interaction is essential to support heterologous synapse formation and the N723S mutant impaired Nrxn-mediated synaptogenesis.

### Mint2 N723S impairs spontaneous excitatory synaptic transmission

**Figure 5:**
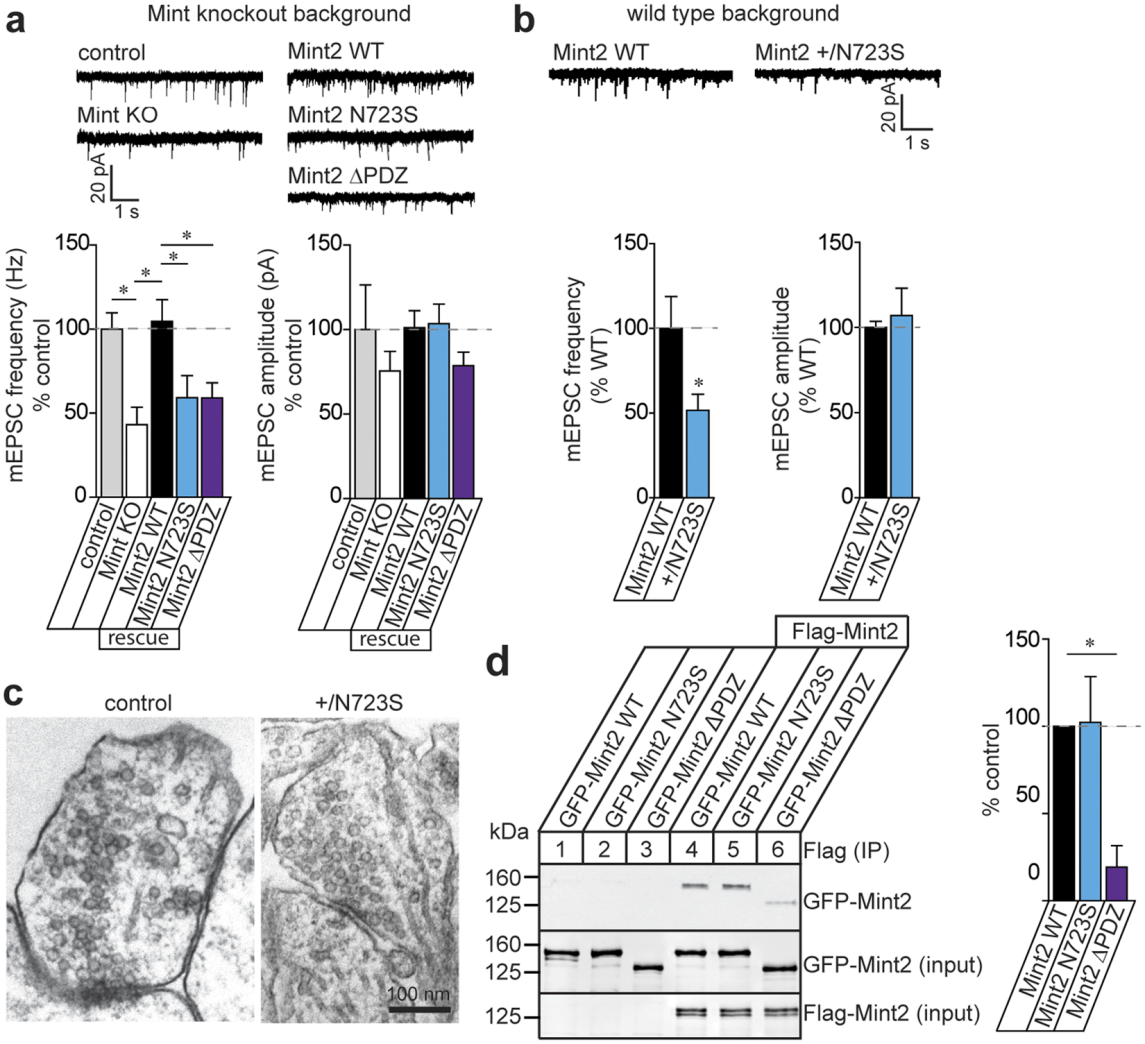
Mint2 N723S decreases spontaneous synaptic activity at excitatory synapses. **(a)** Sample traces showing mEPSC of control, Mint KO or Mint KO rescue with Mint2 WT, N723S, or ΔPDZ mutants at 14–17 DIV. Bar graph of mEPSC frequency expressed as percent control revealed a decrease in miniature frequency in Mint KO neurons compared with control that was rescued by Mint2 WT but not N723S or ΔPDZ mutants. Bar graph of mEPSC amplitude showed no change. Control, n = 9; KD, n = 9; WT, n = 15; N723S, n = 13; ΔPDZ, n = 12 neurons analyzed. “n” represents multiple cells from 4–8 coverslips per condition and analyzed from three independent experiments/cultures. **(b)** Sample traces showing mEPSC of Mint2 WT or N723S mutant in hippocampal WT neurons. Bar graphs expressed as percent control showed that the N723S mutant decreases the frequency but not the amplitude of mEPSC compared to the WT. WT, n = 7; N723S, n = 5 neurons analyzed. “n” represents multiple cells from 4–6 coverslips per condition and analyzed from one independent experiment/culture. **(c)** Ultrastructural analysis by electron microscopy of synaptic structure in hippocampal neurons infected with control or Mint KO neurons rescued with Mint2 +/N723S at 14 DIV. Representative images of asymmetric excitatory synapses. Scale bar, 100 nm. **(d)** HEK293T cells were co-transfected with Flag-Mint2 WT and GFP-Mint2 WT, GFP-Mint2 N723S or GFP-Mint2 ΔPDZ. Cell lysates were collected 48 hour post-transfection and immunoprecipitated (IP) with Flag antibody and immunoblotted for GFP. The results were quantified as relative to the immunoprecipitated level of Mint2 WT (lane 4). The representative images from n = 3 independent experiments were cropped and shown in this figure. Whole gel images are presented in Supplementary Fig. S2. Data represents as means ± SEM and statistical significance was evaluated using one-way ANOVA for (**a,d**) and unpaired Student’s *t-*test for (**b**). All determinations, *p < 0.05 (see text).

To determine whether the synaptogenesis changes in Mint2 N723S neurons led to functional alterations in synaptic transmission, we performed whole-cell recordings to monitor the spontaneous miniature excitatory postsynaptic currents (mEPSCs) in Mint KO neurons rescued with Mint2 WT, N723S or ΔPDZ. We found consistently that Mint KO neurons decrease mEPSC frequency without a change in amplitude compared to control-infected neurons that contain Mint2 [one-way ANOVA, F_(4, 51)_ = 4.174, p = 0.05] (Fig. 5a)^16^. In addition, lentiviral infection with Mint2 WT was able to rescue mEPSC frequency to control levels in Mint KO neurons [one-way ANOVA, F_(4, 51)_ = 4.174, p = 0.8360]. However, N723S and ΔPDZ were not able to rescue the mEPSC frequency back to WT levels [one-way ANOVA, F_(4, 51)_ = 4.174, p = 0.021 for both], indicating a potential impairment in excitatory presynaptic transmission or synapse number (Fig. 5a). Furthermore, we tested whether expression of N723S in wild type neurons produced a phenotype that mimics the Mint KO neurons. Indeed, we found a similar reduction of mEPSC frequency but not amplitude with the Mint2 N723S mutation on an endogenous wild type background. The fact that Mint2 N723S impaired spontaneous mEPSCs on a wild type background supports the notion that Mint2 N723S may act in a dominant-negative manner [unpaired Student’s *t-*test, p < 0.0435] (Fig. 5b).

Previous electron microscopy analysis revealed normal synaptic ultrastructure of presynaptic terminals in Mint deficient neurons^16^. Interestingly, when we analyzed the synaptic ultrastructure of excitatory hippocampal synapses in heterozygous Mint2+ /N723S rescued neurons, we found the number of synaptic vesicles docked at the active zone was decreased by 1.75-fold [unpaired Student’s *t-*test, p < 0.008] while the overall number of synaptic vesicles and the length of the postsynaptic density were unchanged (Table 1). Coinciding with the decreased number of docked vesicles, we also observed a significant decrease in both presynaptic terminal area and circumference in neurons expressing Mint2 +/N723S mutation [unpaired Student’s *t-*test, p < 0.0001] (Fig. 5c and Table 1). Altogether, these ultrastructural excitatory presynaptic changes may be a consequence of decreased vesicle fusion with the plasma membrane and could be a cause for the functional deficit in spontaneous synaptic transmission in Mint2 +/N723S expressing neurons.

**Table 1:** Quantitative ultrastructural analysis of hippocampal synapses.

Parameter	control	Mint2 +/N723S	p-value
Bouton area (μm)	4.03 ± 0.48	2.31 ± 0.16	5.6 E-05***
Bouton circumference (μm)	4.41 ± 0.34	3.23 ± 0.14	4.3 E-04***
PSD length (μm)	0.28 ± 0.04	0.29 ± 0.02	0.711
Docked vesicles/AZ	2.15 ± 0.25	1.23 ± 0.18	0.008*
Docked vesicles 150 nm from AZ	6.85 ± 0.92	6.91 ± 0.48	0.944
Vesicles/terminal	45.08 ± 7.29	39.06 ± 3.34	0.397

All data are presented as means + SEM. Control, n = 16; Mint2 **+**/N723S, n = 35. “n” is the number of synapses from three different coverslips of a single culture. *Indicates significance.

How might Mint2 N723S disrupt the function of the wild type gene? One possibility is that Mint2 dimerizes and the N723S mutant can impair the dimer complex thereby acting in a dominant-negative manner. To test this, we co-transfected Flag-Mint2 WT with GFP-Mint2 WT, GFP-Mint2 N723S or GFP-Mint2 ΔPDZ in HEK293T cells and performed immunoprecipitation assay (Fig. 5d and Supplementary Fig. S2). We found both GFP-Mint2 WT (lane 4) and GFP-Mint2 N723S (lane 5) co-immunoprecipitated with Flag-Mint2 WT showing that Mint2 proteins dimerize and it is unlikely that the N723S mutant is impairing formation of the dimer complex. In contrast, GFP-Mint2 ΔPDZ (lane 6) exhibited a greatly reduced binding to Flag-Mint2 WT [one-way ANOVA, F_(2, 3)_ = 7.947, p = 0.0424], indicating that the PDZ domains of Mint2 are necessary for Mint2 dimerization.

## Discussion

Here, we investigated the cellular and molecular mechanism underlying the Mint2-Nrxn1α interaction and the functional defects of the Mint2 N723S mutant. We showed the N723S mutation profoundly affected Nrxn1α stabilization and trafficking to the membrane without affecting Nrxn1α interaction compared to Mint2 WT (Fig. 1). Our live-cell imaging data in primary mouse neurons demonstrated that the N723S mutation had a greater fraction of immobile puncta which affected axonal trafficking of Nrxn1α to the presynaptic membrane essential for normal synapse formation (Figs 2 and 3). Indeed, we found that the N723S mutation decreased synaptogenesis in an Nrxn-mediated heterologous synapse formation assay (Fig. 4). Lastly, we showed that the N723S mutation caused a decrease in mEPSC frequency without a change in amplitude indicating impairment in presynaptic release machinery that is most likely due to the decrease in the number of docked vesicles (Fig. 5). Overall, our data reveal Mint2 N723S leads to impairments in synaptic formation and function at least, in part, by altering Nrxn1α membrane trafficking in excitatory synapses.

Our data support a role for Mint2 in forming a stable complex with Nrxn1α in the secretory pathway and facilitating surface transport of Nrxn1α and that the Mint2 N723S mutation interferes with this pathway. First, we show Mint2 interacts with the C-terminal sequences of Nrxn1α which is partially mediated by Mint2 PDZ domains. Mint2 increased the total levels of Nrxn1α protein and enhanced Nrxn1α surface levels in HEK293T cells suggesting that Mint2 associates with Nrxn1α and facilitates stability and trafficking. The Mint2-Nrxn1α interaction is critical for Nrxn1α surface trafficking because co-expression of Nrxn1α and Mint2 ΔPDZ mutant disrupted Nrxn1α transport to the cell surface and sequestered Nrxn1α to a cytosolic fraction. Second, we show Mint2 is predominantly localized to the Golgi and is a mobile protein which controls polarized trafficking of axonal Nrxn1α and the N723S mutation had more immobile puncta. These findings are supported by previous studies which show Mints function at the Golgi to control polarized trafficking of other axonal membrane proteins such as APP in the mushroom body of *Drosophila*, a brain structure involved in learning and memory^24^. Third, our data indicate that Mint2 is involved in polarized and regulated targeting of Nrxn1α to synapses. We show the presence of Nrxn1α positive transport vesicles co-localized with Mint2 in axons, suggesting Mint2 functions as adaptors to enable Nrxn1α trafficking to mediate synapse formation. Mint2 may not be essentially required for axonal transport of Nrxn1α as other proteins can also transport Nrxn1α such as CASK and Rim1α^26^, but Mint2 could increase the efficiency of the passage and possibly the organization of Nrxn1α at the synapse. Indeed, we found Mint KO neurons impaired Nrxn-mediated heterologous synapse formation, an effect that was rescued with Mint2 WT. However, Mint2 N723S or heterozygous +/N723S failed to rescue heterologous synapse formation suggesting Mint2 interacts functionally with Nrxn1α to stabilize synapse assembly and Mint2 +/N723S may directly impair synaptic formation. Another possibility is that Mint2 is at a key position at the Golgi where they transiently bind to Nrxn1α, facilitating the packaging of Nrxn1α into trafficking vesicles destined for the presynaptic compartment that can be mediated by other PDZ scaffold proteins such as CASK and Rim proteins and the N723S mutation hinders this ability.

Altered synaptic structure and function have emerged as a strong basis for impaired cognition and underlying cause for ASDs, supported by the prevalence of highly penetrant human mutations in genes such as *NRXN1, NLGN3* and *NLGN4, SHANK3* and *CNTNAP2*^6,33,34^. For example, a Neuroligin 4 (NL4) missense mutation (R87W) associated with autism was shown to block NL4 transport to the cell surface and as a result led to a loss of synapse formation and synaptic strength^35^, a phenotype similar to the Mint2 N723S ASD mutant reported here. Our data supports *MINT2* as an autism candidate gene based on its role in synaptic function. Mint2 acts in excitatory synapses and differs functionally from Mint1, which is predominantly present in inhibitory synapses. Both Mints 1 and 2 are important regulators of presynaptic neurotransmitter release and are essential for mouse survival^16,36^. Interestingly, single Mint2 knockout mice display a deficit in social interaction supporting the role of Mint2 in normal emotional and social development^22^. Here, we found Mint2 N723S selectively impaired mEPSC frequency but not amplitude in both Mint-deficient and WT endogenous neurons, suggesting an impairment of excitatory synaptic transmission with Mint2 N723S. The trafficking and functional synaptic deficits of the N723S mutant could also occur through other proteins that Mint2 or Nrxn1α binds to such as Munc18, CASK and Rim proteins^14,26,37^ where additional components of the active zone and/or exocytotic machinery aids in their passage through the secretory pathway to the synapse.

How might Mint2 N723S disrupt the activity of the wild type protein? We considered several possibilities: (1) Mint2 N723S cannot bind to Nrxn1α; (2) Mint2 dimerizes and the N723S mutant can impair the dimer complex thereby acting in a dominant-negative manner. The observation of the same phenotype in synaptic impairments found in N723S for both Mint deficient and WT neurons support the notion that dimerization is a likely a mechanism underlying the dominant-negative activity; or (3) Mint2 N723S retains binding to Nrxn1α and may out compete the Mint2 WT and interferes with Nrxn1α surface trafficking. The key feature we observe is that Mint2 N723S interacts normally with Nrxn1α and does not alter Mint dimerization; however; a single point mutation in Mint2 N723S impaired mobility and ability to traffic Nrxn1α to the presynaptic membrane that correlated with a decrease in spontaneous synaptic transmission.

We have to keep in mind that while there is growing support for *MINT2* related to ASDs, *MINT2* is not considered a strong risk gene associated with ASDs. For example, several independent studies have identified CNVs in *MINT2* associated with autism, but *MINT2* has not been proven to be responsible for the abnormal phenotype in any of these CNV-associated disorders^6,7^. In addition, the seven nonsynonymous coding variants in *MINT2* that have been identified in rare individuals with ASDs, six of these autism-specific variants including MINT2 N722S for which family members’ DNA was acquired were all inherited from unaffected parents^8^. However, our study spotlights the first functional study of a single mutation in Mint2 that has significant impact on synaptic function. Recently Mint1, structurally similar to Mint2, was identified as one of the 52 genes linked to human intelligence highlighting the importance of the Mint family of adaptor proteins influencing and shaping intelligence^38^. The validation and mechanistic role of new genes such as *MINT2* associated with ASD will directly lead to a better understanding of the molecular pathways underlying ASD pathogenesis.

## Methods

### Plasmids

Seven ASD Mint2 coding variants were made from rat pEGFP-C3-Mint2 using QuikChange II Site-Directed Mutagenesis Kit (Agilent Technologies 200523). The Mint2 ΔPDZ construct that lacks both PDZ domains (1–547 amino acids) was PCR amplified (PrimeSTAR, Clontech) and cloned into pEGFP-C3 (Clontech 632482). For expression in neurons, pEGFP-C3-Mint2 wild type (WT) and mutants were digested with NheI and BamHI and cloned into the lentiviral pFUW vector individually. All plasmids were verified by sequencing. GST-Nrxn1α expression construct and pSyn5-E-mCherry-Nrxn1α were kind gifts from Dr. Thomas Biederer (Tufts University, Boston, MA) and Dr. Markus Missler (Münster University, Germany), respectively. Tagged plasmids that include Flag-Nrxn1α and mVenus-NL1 were gifts from Dr. Antony Boucard (Avandia Instituto Politecnico Nacinoa, Mexico).

### GST pulldown

GST-Nrxn1α fusion protein that encompassed the cytosolic domain was expressed in the BL21 (DE3) strain of *E. coli* and immobilized with glutathione sepharose beads (GE Healthcare). HEK293T cells (CRL-11268; RRID: CVCL_1926) were transfected with either GFP-tagged Mint2 WT, N723S or ΔPDZ using FuGENE6 reagent (Promega). 48 h after transfection, lysates were solubilized in buffer containing 50 mM Tris-HCl pH 7.2, 1 mM EDTA, 150 mM NaCl, 1% NP-40 and proteinase inhibitors. Lysates were incubated in 4 °C for 30 min and centrifuged at 20,000 *g* for 10 min at 4 °C. The supernatant was collected and incubated with 50% slurry of purified GST-Nrxn1α for 2 h at 4 °C. Beads were washed three times with the solubilization buffer and 2 × reducing sample buffer with 10% β-mercaptoethanol was added. Samples were heat denatured and subjected to Western blotting analysis. Relative quantitation was performed by Licor Odyssey CLx imaging system.

### Molecular dynamics calculation

The NMR structure of human Mint2 PDZ2 (PDB_ID: 3SUZ) was used as a template to generate a rat variant with 92% identical residues. Preliminary molecular dynamics calculations were performed using force field AMBERff14SB without applications of residual constrains in UCSF Chimera (rbvi.ucsf.edu). Structures are rendered by PyMOL (pymol.org).

### Subcellular fractionation

HEK293T cells were transfected using FuGENE6 reagent (Promega) and 48 h were washed with 1 × PBS and stored at −80 °C. Cells were warmed up to 37 °C to induce freeze-fracture of the cell membrane and homogenized in 1 ml of 20 mM Tris pH 8.5, 10 mM EDTA pH 8.0, 1 mM PMSF with proteinase inhibitors. Fifty microliters of the starting “total” lysate was set aside and used for western blot analysis (Fig. 1c). The remaining lysate was centrifuged at 135,000 *g* for 1 h at 4 °C. The supernatant “cytosolic” fraction was removed to a new tube and the remaining pellet was solubilized in 10 mM Tris pH 7.6, 150 mM NaCl, 2% Triton X-100, 2% NP40, 1 mM PMSF and proteinase inhibitors and extract for 1 h at 4 °C. The lysate was centrifuged at 100,000 *g* for 1 h at 4 °C and the supernatant “membrane” fraction was removed to a new tube. Membrane fraction includes plasma membranes and other membranous organelles. Samples were heat denatured and subjected to Western blotting analysis. Relative quantitation were performed by Licor Odyssey CLx imaging system. For Fig. 1d, quantification of membrane Nrxn1α is defined as Nrxn1α protein levels in membrane fraction normalized to a membrane marker, Na^+^/K^+^ ATPase protein level and expressed as percent control (which is defined as GFP co-transfected with Nrxn1α)

### Live cell staining

HEK293T cells were co-transfected with Flag-Nrxn1α together with GFP (control), GFP-tagged Mint2 WT, N723S or ΔPDZ using FuGENE6 reagent (Promega) and 48 h were washed with ice-cold PBS. Cells were blocked with ice-cold 5% goat serum in PBS for 30 min and incubated with ice-cold anti-Flag antibody (Sigma at 1:500) in 5% goat serum/PBS for 30 min. Following ice-cold PBS washes, cell were fixed with 4% paraformaldehyde for 10 min at room temperature. All steps prior to paraformaldehyde fixation were done in 4 °C to prevent internalization of surface Nrxn1α. Cells were then incubated with goat anti-rabbit Alexa 546 (1:500) in 5% goat serum/PBS for 30 min. Following PBS washes, coverslips were mounted with ProLong Gold mounting reagent (Invitrogen) and imaged with a Carl Zeiss LSM700 confocal microscope. Total surface staining was quantified by sum of all slices and maximum projection. Depending on the cell size, 5–7 Z-stacked images were captured and images were converted to the sum of slices using the ImageJ program to quantify fluorescence of the entire volume of a cell.

### Primary neuronal cultures and lentivirus production

Primary hippocampal neuronal cultures were prepared from newborn mice of either sex of C57BL/6 J (JAX: 000664) or conditional knockout mouse line that is homozygous for Mint1 and 3 knockout and floxed for Mint2 (*Mint1*^−/−^; *fMint2//fMint2*; *Mint3*^−/−^). All animal experiments were approved by Boston University Institutional Animal Care and Use Committee. All methods were performed in accordance with relevant guidelines and regulations. Neurons were dissociated with trypsin 10 min at 37 °C, triturated and plated onto matrigel-coated coverslips (BD Bioscience). Neurons were maintained in a humidified incubator with 5% CO_2_ at 37 °C. Recombinant lentiviruses were generated by transfecting HEK293T cells with lentiviral plasmids for Δ (a control vector without *cre* recombinase), *Cre*, Mint2 WT, Mint2 N723S, or Mint2 ΔPDZ with viral enzymes and envelope proteins (pRSV/REV, pMDLg/RRE, and pVSVG) using FuGENE6 reagent. The initial media was changed into neuronal growth media after 8 h of transfection. Lentivirus-containing conditioned media was collected after 48 h, centrifuged at 500 *g* for 10 min at 4 °C to remove cell debris and stored at −80 °C.

### Transient transfection of cell lines and primary hippocampal neurons

HEK293T or COS7 cells plated on 18 mm glass coverslips or tissue culture plates were grown to 70–80% confluency in DMEM (Invitrogen) containing 10% fetal bovine serum (Atlanta Biologicals) and 1% penicillin/streptomycin (Thermo Fisher Scientific). Cells were transfected using FuGENE6 reagent. Cultured hippocampal neurons plated on 18 mm glass coverslips coated with Matrigel were transfected with lipofectamine 2000 reagent (Thermo Fisher Scientific) at 4–6 days *in vitro* (DIV) and experiments were performed between 7–10 DIV (for Nrxn1α axonal transport) or 12–14 DIV (for Nrxn1α presynaptic targeting).

### FRAP and time-lapse imaging

Primary mouse hippocampal neurons on glass bottom plates (*In Vitro* Scientific) were infected with lentiviral Mint2 WT or N723S at 3 DIV and used for FRAP experiments at 14 DIV. Using a confocal Zeiss microscope, a 488 nm diode laser was used to photobleach an area encompassing the Golgi with 100% laser power for 3 consecutive cycles (indicated by red horizontal bar in Fig. 2c). Recovery fluorescence was acquired with 1% laser power and imaged every 5 sec for 170 sec. Analysis was generated by the FRAP module (Zen software, Zeiss; RRID:SCR_013672). For time-lapse imaging, images of neuronal processes expressing GFP-tagged Mint2 proteins were captured every 1 sec for 60 sec in a 37 °C chamber with 5% CO_2_ at 14 DIV. No visible signs of photo-damage were observed because low laser power was used during imaging. Kymographs of straightened processes were created with ImageJ program (US National Institutes of Health) and the resulting kymograph shows distance traveled (x-axis) and time elapsed (y-axis).

### Immunocytochemistry

Primary hippocampal neurons were fixed with 4% paraformaldehyde at room temperature for 8 min, permeabilized and blocked in 10% goat serum, 0.1% saponin in PBS. Cells were incubated with primary antibodies in blocking buffer (10% goat serum in PBS) at 4 °C overnight. Following PBS washes, cells were incubated with goat anti-mouse or goat anti-rabbit IgG secondary antibodies conjugated to Alexa Fluor-546 or −647 (Invitrogen) in blocking buffer for 1 h at room temperature. Following PBS washes, coverslips were mounted with ProLong Gold Anti-fade Mount with DAPI (Invitrogen) and imaged with a Carl Zeiss LSM700 confocal microscope. The degree of colocalization is expressed as percentage overlap with mCherry-Nrxn1 using ImageJ. For example, images were merged for GFP (green) and Nrxn1 (red) to create a composite image while keeping the original images. GFP puncta (green), Nrxn1 puncta (red) and merged puncta (yellow, represents colocalization) were counted individually. Puncta density was calculated by the # of puncta/unit length of the processes.

### Heterologous synapse formation assay

COS7 cells were transfected using FuGENE6 with plasmids expressing mVenus control or mVenus-Neuroligin1 (NL1), trypsinized after 24 h and plated onto 10 DIV primary hippocampal mouse neurons. After 48 h co-culture, cells were fixed with 4% paraformaldehyde and labeled with rabbit anti-synapsin P610 antibody (a kind gift from Dr. Thomas Südhof, Stanford University, CA) and Alexa-546 secondary antibody. Z-stacked images were captured with a Zeiss LSM700 confocal microscope and converted to the sum of slices using the ImageJ. Quantification of synapse formation is expressed as percent control determined by the co-localization (yellow in the merged images) of the presynaptic synapsin (red) with postsynaptic mVenus-NL1 (green) expression.

### Electrophysiology

Spontaneous excitatory synaptic transmission was recorded from hippocampal neurons at 14–17 DIV using whole-cell voltage-clamp techniques in the presence of 1 μM tetrodotoxin, 50 μM D-2-amino-5-phosphonovaleric acid, and 20 μM bicuculline. The pipette was filled with electrode solution containing (in mM): 105 CsMeSO3, 10 CsCl, 5 NaCl, 10 HEPES, 0.2 EGTA, 4 Mg-ATP, and 0.3 Na_2_GTP pH 7.4 (300 mOsm—adjusted with 1 M sucrose). Recordings were obtained using an Axopatch 200 A amplifier and 1440 Digitizer and analyzed with Clampfit Software (Molecular Devices).

### Antibodies

The following antibodies were used for immunoblotting and immunocytochemistry: FLAG (mouse, Sigma F3165, 1:500; RRID:AB_259529), GFP (rabbit; Synaptic Systems 132 002, 1:1000; RRID:AB_887725), GAPDH (mouse; Millipore MAB374, 1:5000; RRID:AB_2107445), GM130 (mouse; BD Biosciences 610822, 1:1000; RRID:AB_398141), mCherry (rabbit; Abcam ab167453, 1:500; RRID:AB_2571870), Mint2 (rabbit; Sigma M3319, 1:1000; RRID:AB_477178), alpha 1 Sodium Potassium ATPase antibody (rabbit; Abcam ab7671, 1:200; RRID:AB_306023), Neurexin-1 (rabbit; Synaptic Systems 175 103, 1:1000; RRID:AB_10697816), SMI-312 (mouse; Abcam ab24574, 1:1000; RRID:AB_448151), Synapsin (rabbit; kind gift from Dr. Thomas S üdhof, P610, 1:500), Synaptobrevin (mouse; Synaptic Systems 104 211, 1:1000; RRID:AB_2619758), alpha-tubulin (mouse; Cell Signaling 3873, 1:1000; RRID:AB_1904178).

### Experimental design and statistical analysis

Experimenters were blind to condition during data acquisition and analysis. All image acquisition were kept constant between coverslips and independent experiments/cultures. In addition, all statistical parameters are presented as means ± SEM. Statistical significance between groups was determined by unpaired Student’s *t-*test to analyze all pairwise datasets and the Dunnett multiple comparison test was used with one-way analysis of variance (ANOVA) for parametric analysis of multiple comparisons conducted using Prism 6 software (GraphPad Software; RRID:SCR_002798). Number of experiments and statistical information are reported on the corresponding figure legends. In figure, asterisks denote statistical significance where *p < 0.05; **p < 0.005 and ***p < 0.0005.

## Supplementary information

Supplementary Information
