# Supplementary material for: A rare autism-associated MINT2/APBA2 mutation disrupts neurexin trafficking and synaptic function
Source: Sci Rep. 2019 Apr 15;9:6024. doi: 10.1038/s41598-019-42635-7 (PMC6465354; doi:10.1038/s41598-019-42635-7)
Supplement: Supplementary file 1 — Supplementary Information [file 41598_2019_42635_MOESM1_ESM.pdf]

A rare autism-associated MINT2/APBA2 mutation disrupts neurexin trafficking and synaptic function

Amy Y. Lin[1], Shawna Henry[1], Carsten Reissner[2], Christian Neupert[2], Connor Kenny[1], Markus Missler[2, 3], Uwe Beffert[1] and Angela Ho[1,*]

[1] Department of Biology, Boston University, 24 Cummington Mall, Boston, MA 02215, USA

[2] Institute of Anatomy and Molecular Neurobiology, Westfälische Wilhelms-University, 48149 Münster, Germany

[3] Cluster of Excellence EXC 1003, Cells in Motion, 48149 Münster, Germany

*Corresponding author: Angela Ho, Department of Biology, Boston University, 24 Cummington Mall, Boston MA 02215, aho1@bu.edu

Supplementary Figure Legends

Supplementary Figure S1. (a) Representative whole immunoblots from Figure 1b of protein levels from total cell lysates of HEK293T cells transfected with Mint2 WT or Mint2 ASD variants. Tubulin served as loading control. Unrelated samples indicate the section of the western blots that do not appear in main text. **(b)** Quantification showing Mint2 mutants did not disrupt Mint2 protein expression compared to Mint2 WT. **(c)** Representative whole immunoblots from Figure 1c of protein levels from total cell lysates of HEK293T cells co-transfected with Nrxn1α and either GFP-tagged Mint2 WT, N723S or ΔPDZ mutant. GAPDH served as loading control. **(d)** Representative whole immunoblots from Figure 1d of protein levels from cell lysates of HEK293T cells co-transfected with Nrxn1α and either GFP (control), GFP-tagged Mint2 WT, N723S or ΔPDZ and subjected to subcellular fractionation. GAPDH and Na^+/K^+ ATPase were used as cytosolic and membrane markers, respectively. **(e)** Representative whole images from Figure 1f of protein levels from cell lysates of HEK293T cells transfected with Mint2 WT, N723S or ΔPDZ bound to GST-Nrxn1α fusion protein. Coomassie stained gel of GST-tagged Nrxn1α protein. Unrelated samples indicate the section of the western blots that do not appear in the main text.

Supplementary Figure S2. Representative whole immunoblots from Figure 5d of protein levels from cell lysates of HEK293T cells were co-transfected with Flag-Mint2 WT and GFP-Mint2 WT, GFP-Mint2 N723S or GFP-Mint2 ΔPDZ. Cell lysates were collected 48 hour post-transfection and immunoprecipitated (IP) with Flag antibody and immunoblotted for GFP. The results were quantified as relative to the immunoprecipitated level of Mint2 WT (lane 4).

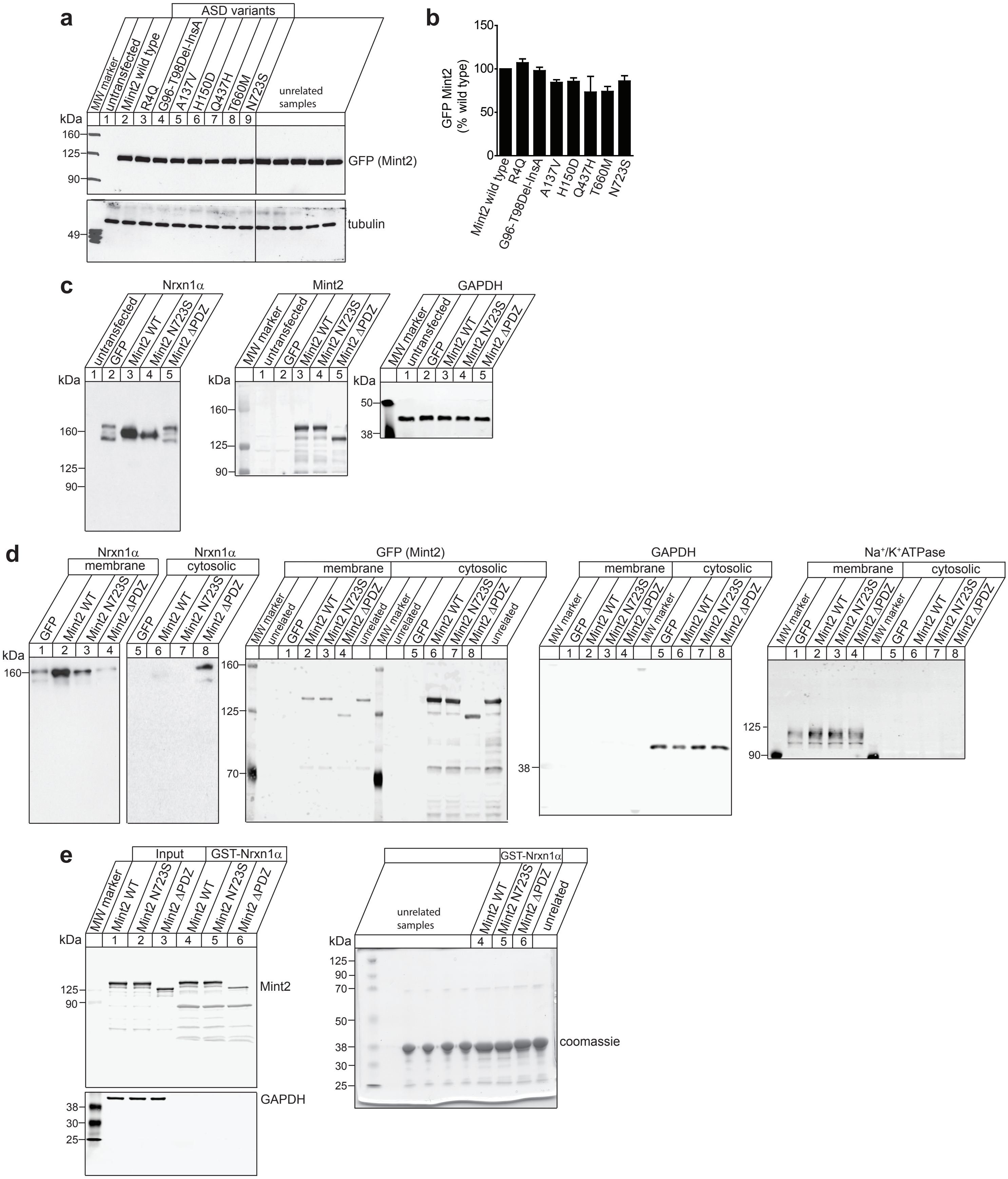
a
ASD variants
MW marker
untransfected
Mint2 wild type
R4Q
G96-T98Del-InsA
A137V
H150D
Q437H
T660M
N723S
unrelated samples
kDa
1 2 3 4 5 6 7 8 9
160
125
90
49
GFP (Mint2)
tubulin
b
GFP Mint2 (% wild type)
150
100
50
0
Mint2 wild type
R4Q
G96-T98Del-InsA
A137V
H150D
Q437H
T660M
N723S
c
Nrxn1α
Mint2
GAPDH
MW marker
untransfected
GFP
Mint2 WT
Mint2 N723S
Mint2 ΔPDZ
kDa
1 2 3 4 5
160
125
90
50
38
d
Nrxn1α
membrane
cytosolic
GFP (Mint2)
GAPDH
Na⁺/K⁺ATPase
MW marker
unrelated
GFP
Mint2 WT
Mint2 N723S
Mint2 ΔPDZ
kDa
1 2 3 4 5 6 7 8
160
125
70
38
90
e
Input
GST-Nrxn1α
MW marker
Mint2 WT
Mint2 N723S
Mint2 ΔPDZ
kDa
1 2 3 4 5 6
125
90
Mint2
38
30
25
GAPDH
unrelated samples
unrelated
4 5 6
125
90
70
50
38
30
25
coomassie

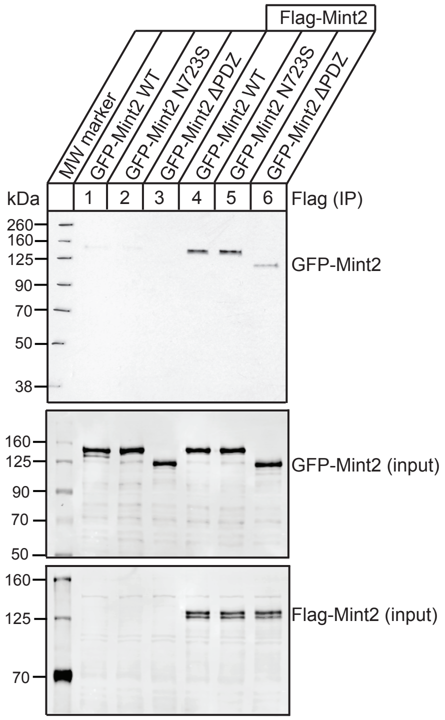

Flag-Mint2
MW marker
GFP-Mint2 WT
GFP-Mint2 N723S
GFP-Mint2 ΔPDZ
GFP-Mint2 WT
GFP-Mint2 N723S
GFP-Mint2 ΔPDZ
kDa
1
2
3
4
5
6
Flag (IP)
260
160
125
90
70
50
38
GFP-Mint2
160
125
90
70
50
GFP-Mint2 (input)
160
125
70
Flag-Mint2 (input)
